# Gonorrhœa and Its Treatment

**Published:** 1898-04

**Authors:** Winslow Anderson

**Affiliations:** A. M., M. D., M. R. C. P. Lond., M. R. C. S. Eng., Etc.; Professor of Gynæcology and Abdominal Surgery, College of Physicians and Surgeons of San Francisco, California


### GONORRHŒA AND ITS TREATMENT.

By WINSLOW ANDERSON, A. M., M. D., M. R. C. P. Lond., M. R. C. S. Eng., Etc.

Professor of Gynæcology and Abdominal Surgery, College of Physicians and Surgeons of San Francisco, California.

The prevalence of gonorrhœa and its frightful ravages are brought to the notice of the gynæcologist almost daily. The gonococcus infects not only the vulva, urethra, bladder, rectum, vagina and uterus but also the Fallopian tubes, ovaries and pelvic peritoneum, and may lie dormant in many of the tissues, notably the vulvo-vaginal, Skene's urethral, cervical and utricular glands, for years, ready to infect or reinfect any healthy mucous membrane. Undoubtedly the prime cause of *sterility* in both man and woman is the gonococcus. One of the great causes of dysmenorrhœa is the gonococcus and the prime ætiological factor of pyosalpinx, pelvic cellulitis, co-ophoritis, and the subsequent salpingo oophorectomy, to say nothing of the cystitis, pyonephritis, can be traced to the gonococcus with its boon companions, the streptococcus and the staphylococcus.

Primary Sterility and Gonorrhœa.—Vedeler in the *Centrabl. f. Gynak.*, No. 26, 1897, who investigated 310 sterile women, according to the *British Medical Journal*, found that undoubtedly gonorrhœa is the most frequent cause of sterility. The average years of marriage in the series were three, the minimum one complete, whilst seventy-two of the women had been married over ten years. Vedeler succeeded in examining fifty of the husbands, and found that thirty-eight had had gonorrhœa and that thirty-four had infected their wives. He calculates that at this rate 235 of the 310 husbands probably had had that disease, and that about 210 must have infected their wives.

Dr. Madden (*London Lancet*) recorded by Dr. Chase in the *Brooklyn Medical Journal* for October, 1896, after speaking of the invasions of gonococci into the uterus, Fallopian tubes, etc., says: "With reference to the other intra-pelvic complaints of which gonorrhœa is a fertile source, I shall only here observe that long clinical experience has convinced me that in a large proportion of instances, peri-uterine phlegmon, or in other words, all these chronic inflammatory lesions of the pelvic serous and connective tissues formerly included in the term pelvic cellulitis, and subsequently better known as perimetritis and parametritis, may be found traceable to that affection."

Endometritis and Pelvic Inflammation due to Gonococci.—Gottchalk and Immewar in *Archiv. fur Gynakol*, bd. i. h. 3, report finding gonococci in several cases of endometritis. Acute infection of the pelvic organs is almost as common from gonorrhœa as from puerperal and other septicæmic causes.—Walter

B. Chase, M. D. (*Brooklyn Medical Journal*, October, 1896). Pelvic inflammations from gonorrhœa are probably of more frequent occurrence than acute orchitis in the male.—Sir Spencer Wells. Dr. F. G Moehlan, of Buffalo (*St. Louis Medical Era*) attributes pelvic diseases in women to the gonococcus in *ninety per cent of all cases*. Prof. Marcus Rosenwasser in the *Cleveland Journal of Medicine* says: "Not less than twelve per cent of all the women who consult the specialist, exclusive of prostitutes, have gonorrhœa or its sequelæ. It is the cause of not less than fifteen per cent. of all the cases of puerperal fever. It is responsible for seventy per cent. of all cases of *sterility in woman*."

Duration of Acute Gonorrhoea.—Dr. Christian, of Philadelphia, writes positively on the duration of acute gonorrhœa under treatment, in the *Therap. Gazette* of January '97, as follows: "For my part I believe, with Taylor, that gonorrhœa may be, and very often is, one of the most formidable diseases that can attack man." What, then, is the duration of acute uncomplicated gonorrhœa? Finger states that cases of acute urethritis with no unusual symptoms last from five to six weeks. In the opinion of R. W. Taylor, a man should consider himself very lucky to be cured of gonorrhœa in from six to eight weeks. Keyes places the average duration at from four to six weeks. Brewer says: "Experience in the treatment of urethral disease has taught me that the simple cessation of discharge by no means indicates a cure of the disease." Dr. J. William White gives the duration of acute urethritis at from four to six weeks. Dr. Martin thinks a case fortunate that is cured in eight weeks. The gonococcus thrives better in an acid than in an alkaline medium, hence passing urine after a questionable exposure would aid the multiplication of the germs. The normal urethra is alkaline or neutral, but never acid (Jadassohn), even after passing acid urine, as the canal secretes an alkaline mucus.

Gonorrhœa and Prostatism.—Eraud calls attention to the fact that failure of treatment, in some cases of enlarged prostate in elderly men, may depend not infrequently on gonorrhœal infections, either ancient or recent.—Jour. Cut. and Gen. Urin. Dis.

How to Find the Gonococcus.—Prof. J. A. Wyeth of New York, writes in the December *Polyclinic:* " Place a small drop of the discharge upon a cover-glass and smear by rubbing two cover-glasses together; dry it by passing one of the cover-glasses with pus side upward through a spirit-flame two or three times; immerse this at once in a solution of methyl-blue; wash off the excess of coloring matter by holding it under clean running water, or by dipping the glass several times into clean water; dry the stained pus well by pressing with blotting paper; then cover it with a small drop of cedar oil; put on a thin cover-glass and examine with a lens magnifying from 700 to

1,000 diameters. The peculiar double beam-shaped arrangement of the diplococci will be seen within the protoplasm of the pus corpuscle and epithelium."

Prevention of Gonorrhœa.—Von Neisser (*Centralbl f. d. Krankh. d. Hard. und Sex. Org.*, bd. vii, h. 1, 1896; quoted from *Berl. Klin Woch.*, 36, 1895) has theoretically confirmed the method of preventing gonorrhœa advocated by Blokusewski-Daum. This method consists in dropping into the meatus, the lips of which are held open, two drops of a ten per cent. solution of nitrate of silver. One drop is allowed to run over the frenum. Fifty patients adopted this treatment without experiencing the least irritation. Five seconds' application of this solution always inhibited the growth of the gonococcus when cultivated in artificial media. Weaker preparations are unreliable.—*Therap. Gazette*, July, 1896.

Gonorrhœa of the Rectum.—According to Baer (*Am. Jour. Surg. and Gyn.*) gonorrhœa may and often does invade the rectum in women, from (1) abnormal sexual intercourse, (2) indirectly from the communication with the rectum of an organ attacked with gonorrhœa. By the conveyance of the germs from without by pus, or by therapeutic measures (thermometry, irrigation, etc.). When developed, some patients who have ulceration complain of marked pain on defecation; others discharge both pus and blood via ano. There is usually a slight discharge, which always contains typical gonococci. The treatment consists in injection of a 3 per cent. solution of boric acid, and to diminish the secretion and destroy the gonococci a solution of nitrate of silver 1–3000, employing a quart of each solution. The treatment should be continued until no germs can be detected in the discharge, usually for from five to six weeks.

Toxicosis of Gonorrhœa.—Gonorrhœal ptomainæmia, or the absorption of the alkaloidal poison produced by the gonococcus, is no doubt responsible for our so-called gonorrhœal rheumatism and some of our cases of septic endocarditis. The Brit. Med. Jour., under the heading of Gonococcosis, states that Dr. De'Aulnay, in a reprint from Rev. Internat. de Med. et de Chir., December 10th and 25th, 1896, publishes an exhaustive pamphlet on the diseases produced by the gonococcus. He distinguishes the local and general effects of the micro-organism under the names of ganococcitis and gonococcis, appyiling that of gonococcosis to the whole subject. He points out that the local effects are produced by the growth of the diplococcus mainly upon the mucous membranes, where it brews a toxin which can be absorbed by the circulatory stream. The micro-organism rapidly invades the tissues, particularly the most friable, the cylindrical and serous epithelia, and spreads to the adjoining mucous membranes either by direct cellular infection or by means of the superficial lymphatics. The author divides the general effect of the virus into two categories. First comes the action of the toxins, which is mainly expended upon the blood, the kidneys, the nervous system, the skin, and the serous membranes, especially of the joints. Secondly, there is the action of the micro-organisms themselves, which are conveyed in the blood vessels and lymphatics, either separately, enveloped in leucocytes, or entangled in emboli, which manifest their power of rapid growth when in contact with serum. They tend to the production of pus, which is sterile if the gonococcus is alone, infectious if accompanied by other organisms. In the worst cases, without obvious cause, gonorrhœal pyæmia may develop.

Treatment of Gonorrhœa.—Gonorrhœa is essentially a local disease of the mucous membranes depending upon specific pathogenic infection. When this germ is attacked early by appropriate remedies the disease can be aborted. When allowed to "run its course" as a "self-limited disease," we must not be surprised to find septic or alkaloidal systemic poisoning, nor any of the many serious pelvic sequelæ. In many cases of chronic gonorrhœa that have been treated for months and apparently cured, I have found that Skene's glands, Bartholin's glands, the cervical and utricular glands contained the gonococcus snugly hidden away, ready on the slightest provocation to infect, or reinfect, any mucous membrane.

When we remember the pathological process induced by the gonococcus it is clear that our first object should be to destroy the pathogenic germ. For this purpose any of the following germicides may be used:

| | |
|---|---|
| Permanganate of potassium | 1–10,000 to 1–500 |
| Formalin | 1-1,000 to 1–500 |
| Chinosol | 1–10,000 to 1–1,000 |
| Bichloride of mercury | 1–10,000 to 1–500 |
| Peroxide of hydrogen | 1–4 or 1–2 |
| Argentamin | 1–10,000 to 1–500 |
| Thymol | 1–1,000 to 1–100 |
| Oxycyanatum | 1–1,000 to 1–500 |
| Nitrate of silver | 1–100 to 1–10 |
| Carbolic acid | 1–100 to 1–20 |
| Lysol | 1–100 to 1–20 |
| Argonin | 1–50 to 1–10 |
| Creolin | 1–100 to 1–50 |
| Resorcin | 1–100 to 1–50 |
| Creosote | 1–100 to 1–50 |
| Zinc chloride | 1–50 to 1–10 |
| Saturated sol. salicylic acid in alcohol | 1–10 |
| Acetate of lead | 1–100 to 1–50 |
| Sulphate of zinc | 1–100 to 1–10 |
| Iodoform—powder or ointment | |
| Chloral | 1–50 to 1-10 |
| Ichthyol | 1–50 to 1–10 |
| Actol | 1–1,000 to 1–100 |
| Itrol | 1–1,000 to 1–100 |
| Cresol | 1–1,000 to 1–100 |
| Chlorine water | 1–100 to 1–50 |
| Turpentine | 1–100 to 1–20 |
| Labarraque's solution | 1–50 to 1–10 |

| | |
|---|---|
| Iodine | 1–100 to 1–50 |
| Alum | 1–50 to 1–10 |
| Boric acid | 1–50 to 1–10 |

Neisser, the discoverer of the gonococcus, who has made a special study of the disease, stated at a recent discussion in the Gyn. sect. of the Sixty-eighth Congress of Ger. Natur. and Phys., held at Frank. am Main (Archiv. f. Der. und Syph.), and quoted in the Therap. Gazette of January, 1897, that the medicaments of choice are the salts of silver, such as: Argentamin, nitrate, actol, itrol, bichloride of mercury, oxycyanatum and ichthyol. The remedy must be brought in contact with every portion of the infected mucous membrane and should be used as early as possible. Saenger (Ibid) states that the exact period during which gonococci may persist is not known. He describes vulvitis, adenitis, urethritis, endometritis, metroendometritis, salpingitis and nearly all the inflammatory diseases of the vagino-uterine tract as due to residual gonorrhœa. Baumm (Ibid) claims that infection from an old case when planted on healthy mucous membrane produces an acute attack. He states that gonococci may remain virulent in the genital tract from five to ten years.

Abortive Treatment of Gonorrhœa: Permanganate of Potassium.—In the Ann. d. Mal. d. Org. Gen.-noin, 1896, Dr. J. Janet, as reviewed by Dr. Swinburne in the Jl. of Cut. and Gen.-Urin. Dis. for February, 1897, says, in giving his reasons for using the permanganate of potassium treatment, that in "'every case' in which he has used the treatment before the acute symptoms have manifested themselves, among which swelling of the urethral mucous membrane is the most important, he has been able to attain absolute suppression of the gonorrhœal process, and from the beginning of the treatment up to the time of complete cure not one of the patients showed any increase of invasion." Dr. Vigueron of Marseilles has employed Janet's permanganate treatment for five years with excellent success, curing patients that came under treatment within the first thirty-six hours in from six to ten days. Dr. Guiard of Paris has found Janet's method superior. He uses the permanganate fluid in milder solutions, varying in strength from 1–10,000 to 1–6,000. He reports curing cases with six to twelve irrigations. Dr. Nouges of Paris uses the Janet method for all acute cases (before the fourth day after infection). He uses permanganate lavage in 1–4,000 up to 1–500, the latter for the anterior urethra only. The irrigation is used at first every twelve, eighteen and finally every twenty-four hours. Dr. Nogues has also found nitrate of silver 1–2,000 and oxycynate of mercury efficacious in cases in which the permanganate failed. Dr. Evand of Lyons uses, besides the permanganate, bichloride of mercury, methyl blue, or gentian violet in weak solutions. Dr. Desnos of Paris employs permanganate as follows: He begins with a 1 in 500 solution for the anterior urethra. In twelve hours he uses a 1 in 1,000 solution. On the third, fourth and fifth days he uses a 1 in 5,000. Thus employed within the first thirty-six hours after infection he has cured seventeen out of eighteen cases; the gonococci disappearing after first, second or third lavage. Dr. Janet believes in using weak solutions of the permanganate, 1–10,000 to 1–5,000. He employs irrigation of the entire canal. In five years he had not observed a single case of stricture among the cases treated by this method. Dr. Christian of Philadelphia obtained the best results by daily irrigation combined with the internal administration of sandalwood oil and balsam of copaiba. He uses: astringent injections of zinc ascetate or lead acetate, gr. iii to ℥i or copper sulphate of zinc chloride, gr. one-half to ℥i. His favorite is permanganate of potassium, 1–4,000 to 1–5,000.

Methyl Salicylate in the Treatment of Gonorrhœa.—By reason of the great power possessed by methyl salicylate of penetrating investing membranes, M. Duquaire (cited in the Jl. des Pract.), has conceived the idea that it will reach the gonococci even when they are seated in the deepest layers of the mucous membrane. He reports a case in which its employment cured the disease in five days. He uses the following solution: Methyl salicylate, 1 part; bismuth subnitrate, 20 parts; liquid vaseline, 100 parts. Three injections are to be given daily. The patient should urinate, then take the injection and hold it in the urethra as long as possible. The injections are not painful—N. Y. Med. Journal; Cin. Lancet Clinic.

The Use of Formalin in Gonorrhœa in Women.—In the Jour. de Med. de Paris of August 16, 1896, De Smet calls attention to the treatment of gonorrhœa in women by means of formalin. The vulva is washed with a 1–1,000 solution of formalin, and before introducing the speculum the vagina is washed out with a two or three per cent. solution. It may also be well to swab out the cervical canal of the uterus by the use of a 2-1,000 solution. Where the case is grave and there exists a gonorrhœal metritis, surgical intervention, such as curettage, is needed; but even in these cases the formalin applications are of advantage. De Smet believes that formalin offers the most rapid available means of cure of gonorrhœa in the female.—Therap. Gazette.

Argonin in Gonorrhœa.—Dr. Daniel, of Texas, reports in the Am. Therapist for Jan., 1897, the treatment of twenty-six cases of acute gonorrhœa. "Argonin is a combination of silver with the casein of milk." From 3 to 10 per cent. of the argonin in water was used per injection. The gonococci disappeared in from the second to the seventh day, and a complete cure of the disease was effected in from twelve to eighteen days.

Treatment of Urethritis by Ichthyol and Blue Ointment.—A. T. Ilinsky. (Wratch. Nos. 1, 2, 6, 1896.) With this treatment fourteen acute and fifty-eight chronic cases were treated with good results. He uses a 1 or 2 per cent. solution of ichthyol as an injection twice daily. In chronic cases the following was of great service:

Ung. ciner........................℥ss

Adipis lanæ.

Ung. paraffini.............aa, ʒii

A soft bougie (No 10–15) covered with this ointment is introduced every second day and left in place for twenty-five to thirty minutes. Ichthyol suppositories, eight centimeters in length (one grain in each), produce a striking effect. Visible improvement is seen in cases of prostatitis and other local urethral inflammations from the use of bougies of gray ointment.—Jour. Cut. and Gen. Urin. Dis.

Vaginal Douching.—Dr. Newman of Chicago, quotes Dr. Ellis of Edinburgh (Lon. Lan., May, 1897), in Medicine, July, 1897, on the choice of a solution. The douching fluid is divided into three kinds: 1, neutral or aseptic; 2, mild antiseptic; 3, powerful antiseptic. The first comprises boiled water, alum, ʒi to the pint; acetate of lead, ʒi to the pint; chloride of lead ʒi to the pint; permang. of potassium, weak solution, 1–5,000 to 1–10,000. 2. Mild antiseptic: Boric acid, ʒii to Oi; sulphocarbolate of zinc, ʒii to Oi; ac. carbol, 1–80 to 1–40; Condy's fluid, ʒii to Oi; cresol, 1–250; lysol, 1–250; bichloride, 1–5,000; chinosol, 1–8,000. 3. Powerful antiseptics: Ac. carbol, 1–40 to 1–20; iodine liniment, ʒi–ʒi to Oi; bichloride, 1-1,000 to 1-4,00; chinosol, 1–1,000 to 1–8,000.

Creosote used in Gonorrhœa.—According to the Brit. Med. Jour. for July, 1896, Asmus Meditzinskoie Obozrenie reports 58 male cases of acute gonorrhœa treated by 1--1,000 to 1--100 emulsion of creosote injections. The patients recovered more rapidly than by any other form of treatment and complications were rare.

Sodium Copaibate.—Martin (Phila. Polyc.) has been treating a number of cases of acute and chronic gonorrhœa by the administration of sodium copaibate. Five, ten, fifteen and twenty grains are given four to six times a day, and so encapsulated that they are not set free until they reach the intestinal tract.—Amer. Jour. Surg. & Gyn.

Sodium Salicylate and Balsam for Gonorrhœal Rheumatism. —Balzer (Ther. Gazette, Oct., 1896) recommends large doses (45 to 60 grains) of sodium salicylate with some balsam in the acute, and potassium iodide in the chronic form of gonorrhœal rheumatism. He immobilizes the limb, uses ice bags to painful spots and applies an ointment composed of:

Acidi salicylatis...

Olei terebinth......aa ℨiiss.

Lanoline.............

Adipis..................aa q. s. ad. ℥iij.

M. et ft. ung.

Guaiacol ointment ℨi to ℥i is also employed. In chronic cases, counter-irritation, massage, electricity and turpentine baths are used. In effusion he uses compression. If purulent, he aspirates and injects ℨi--ℨij of a 1--4,000 bichloride solution. If inflammation is severe arthrectomy is recommended.

The Treatment of Gonorrhœa in Women.—The plan of treatment which I have found most satisfactory is as follows: When the case comes under observation within the first thirty-six hours after contagion I swab out the vagina thoroughly with a 1 in 500 solution of potassium permanganate. By dilating the vaginal canal thoroughly with a wire speculum the remedy reaches all the sulci between the rugæ. I next apply the same remedy to the cervical canal and to the urethral glands and insert a vaginal tampon against the cervix soaked in a solution of 1 in 5,000 of permanganate of potassium in boro-glycerine. The patient is then sent to bed and a liberal complement of blue mass given. The next morning ℥ii of a saturated solution of magnesium sulphate in aromatics is exhibited. Every six hours after the first application a douche is given of 1 in 1,000 permanganate of potassium in one gallon of sterilized water at a temperature of 110 deg. F., applying after each douche the permanganate and glycerine tampon. This treatment is continued for twenty-four hours, after which the douches are administered every eight to twelve hours, and the strength of the permanganate is reduced to 1 in 2,000 or 1 in 10,000, continuing the glycerine tampons with or without the permanganate, ichthyol, chinosol or any other antiseptic best suited. The diet must be bland and non-irritating, and large draughts of demulcents administered.

With these simple measures the extension of the disease into the uterus or urethra is prevented, the gonococci are destroyed and the case is generally cured in from five to ten days. When the disease is sub-acute or chronic, which it becomes in two to six weeks, these simple measures will not suffice. If the urethra and Skene's urethral glands are infected it is necessary to apply the antiseptic more frequently to these parts. For this purpose I often use peroxide of hydrogen, 1 to 4 of Marchand's medicinal strength. When the vulvo-vaginal glands are involved, as they are liable to be after the first week or two of the disease, I catheterize the ducts and inject them with permanganate or peroxide every day by means of a small canula. Should the cervical and utricular glands be infected, as they are apt to be in a week or two of untreated gonorrhœa, I know of no better treatmant than a thorough curettement, followed by the intra-uterine permanganate douche, 1-10,000, and the application of equal parts of iodine and carbolic acid. The cavity is then packed with sterilized gauze to assure free drainage. The packing is removed in twelve hours, and the intra-uterine douches continued for two or three days. The hot antiseptic vaginal douches of permanganate, 1 to 10,000 to 1 to 1,000, are used twice daily. The bowels are kept relaxed by salines. Meat, spices, wine, beer and spirits are forbidden. When the urethra and bladder become infected I frequently wash them out daily with a 1 in 10,000 permanganate solution or a boric acid solution. In the sub-acute and chronic stages sandalwood oil and the balsams, with the acetates and citrates of sodium or potassium, are serviceable. Anodynes, such as hyoscyamus, the bromides or chloral, may be required.

Our efforts should be first directed towards the killing of the coccus, as well as towards preventing the extension of the disease into the uterus and Fallopian tubes, which I believe can be accomplished by the methods I have described, if the case is seen within the first three days. When the disease has reached the uterine cavity we can not do better than treat it vigorously to prevent its entrance into the tubes, and the curette is followed by the best results in my hands. Even after the gonococcus has entered the tubes it cannot thrive there, and if there is no fresh infection from the uterus much damage may be prevented by the early use of the curette and intra-uterine medication, as the gonococcus can not live in the abdominal cavity. The staphylococcus and the streptococcus, however, which frequently accompany the gonococcus, seem to flourish both in the tubes and pelvic cavity, inducing the many serious sequelæ which the gynæcologist is so frequently called upon to treat.

My experience with chinosol has been highly satisfactory. Likewise the bichloride used thoroughly and systematically has proven itself efficient, although at times setting up considerable irritation, which the permanganate does not do to the same extent. Formalin is a valuable antiseptic. So are many of the other remedies. I consider it of importance to use antiseptic tampons of any of the established germicides in order to prevent the entrance of the coccus into the uterus, as well as to keep the walls of the vagina apart. I also use an antiseptic vulva pad, soaked either in the permanganate or bichloride solutions, to prevent the urethra, vulvo-vaginal glands and rectum from becoming invaded. In the acute cases the patient is kept in bed three days. In the chronic cases a thorough curettement and antiseptic treatment, with two weeks' rest in bed, have been most successful in my hands. Thorough systematic treatment (which is not readily submitted to by patients) is the sine qua non of an early cure or a possible prevention of the frightful ravages of this disease.—Pacific Medical Journal.

